# Stereotactic Ablative Radiotherapy Fractionation for Hepatocellular Carcinoma in the United States

**DOI:** 10.7759/cureus.8675

**Published:** 2020-06-17

**Authors:** Niki Sheth, Virginia Osborn, Anna Lee, David Schreiber

**Affiliations:** 1 Radiation Oncology, State University of New York - Downstate Medical Center, New York, USA; 2 Radiation Oncology, NYC Health + Hospitals/Elmhurst, New York, USA; 3 Radiation Oncology, Memorial Sloan Kettering Cancer Center, New York, USA; 4 Radiation Oncology, Summit Medical Group, Berkeley Heights, USA

**Keywords:** stereotactic body radiotherapy, stereotactic ablative radiotherapy, hepatocellular carcinoma, hcc, sbrt, sabr, radiation therapy, radiation fractionation, practice patterns, radiation

## Abstract

Introduction

This study aims to analyze the patterns of care, including fractionation and utilization, of hypofractionated stereotactic ablative radiotherapy (SABR) in the treatment of hepatocellular carcinoma (HCC).

Methods

The National Cancer Database was queried for patients diagnosed with HCC from 2004 to 2014 and treated with SABR in three, four, or five fractions in 15-20Gy, 10-13Gy, or 6-12Gy per fraction, respectively. Patients with stage IV and Charlson-Deyo Comorbidity Index > 0 were excluded in order to avoid bias resulting from the selection of poorer prognosis patients. The patients were then stratified based on several characteristics including biologically equivalent doses (BEDs) of =/> 100 Gy and <100 Gy to determine whether there was an association with overall survival (OS) and a multivariable analysis (MVA) was performed to assess for potential confounding factors.

Results

There were 462 patients identified in whom the most common SABR fractionation regimen was 10Gy x five fractions (25.3%), followed by 8Gy x five fractions (17.7%), and 15-16Gy x three fractions (26.4%). A total of 152 patients were treated to a BED < 100Gy, which was associated with a median OS of 20.8 months (95% CI 14.55-27.11). Three hundred and ten patients were treated to a BED =/> 100Gy, which was associated with a median OS of 30.8 months (95% CI 5.25-32.08). On MVA, BED =/> 100Gy was not significantly associated with improved OS (HR 0.85, 95% CI 0.64-1.14, p = 0.28). Factors that were associated with significantly worse survival were tumor size in the largest quartile (HR 2.197 CI 1.440-3.354, p < 0.0001) and T3a disease (HR 2.474 CI 1.472-4.158, p = 0.001 compared to T1).

Conclusion

SABR fractionation schemes vary widely, but are most commonly 10Gy x five fractions followed by 8Gy x five fractions and 15Gy x three fractions. BED of at least 100Gy is not associated with improved OS. Further studies are needed to best identify the optimal SABR dose and fractionation.

## Introduction

Of all malignant cancers arising in the liver, primary hepatocellular carcinoma (HCC) accounts for approximately 90% of them [1]. Though the incidence rates of HCC in the United States were historically lower than in many of the developing countries, they have recently doubled in recent decades, thought to be due to increasing Hepatitis C and nonalcoholic fatty liver disease (NAFLD) incidence [2-4]. 

Treatment of patients with HCC is complicated for several reasons, including different etiologies of HCC and their impact on treatment response, multiple available treatment options, and the potential presence of underlying liver disease. For a great majority of these patients, surgical resection or liver transplant, though the standard of care, are not ideal options due to poor liver function, macrovascular invasion, large bulky tumors, and/or significant co-morbidities. For this patient population, non-surgical, local treatment options utilizing interventional radiology procedures and radiation therapy play a critical role. 

One such local therapy option is external beam radiation therapy (EBRT). In prior years, radiation played a limited role in the treatment of HCC due to low hepatic radiation tolerance. However, this role has expanded significantly in recent years with increasing experience, improvement in existing RT techniques, and development of new methods of delivering RT such as hypofractionated stereotactic ablative radiotherapy (SABR). There has been growing evidence for the use of SABR for patients with unresectable, recurrent, or locally advanced HCC which has, in turn, lead to increasing use and inclusion in the National Comprehensive Cancer Network Guidelines as a category 2b option for inoperable and ineligible for transplant tumors [5-8]. In this study, we used a national database to analyze the patterns of care, including fractionation and utilization, of SABR in patients with locally advanced, unresectable, or recurrent HCC.

## Materials and methods

The National Cancer Database (NCDB) is a nationwide oncology outcomes database created by the American Cancer Society and the Commission on Cancer of the American College of Surgeons. It collects information on approximately 70% of all new invasive cancer diagnoses in the United States by the over 1,430 participating hospitals. The Commission on Cancer’s NCDB and the participating hospitals are the source of the de-identified data used in this study. However, they have not been verified and are not responsible for the statistical validity or conclusions derived by the authors of this study. Exemption was obtained from the New York Harbor Veterans Affairs Committee for Research and Development prior to the initiation of the study. 

We queried the NCDB for patients diagnosed with HCC (histology code 8170-8175) 2004-2014. Of those, we selected for patients treated with SABR by identifying patients treated with radiotherapy in three to five fractions for whom total dose was known. In order to determine the most likely used ablative doses, SABR was further defined as 15-20Gy per fraction for three fractions, 10-13Gy per fraction for four fractions, or 6-12Gy per fraction for five fractions. Doses per fraction that were not divisible by 50 or 100 (e.g., 668 cGy) were excluded. Additionally, in order to avoid bias resulting from the selection of poorer prognosis patients, those with higher Charlson-Deyo Comorbidity Indices and stage IV disease were also excluded.

Patient characteristics were stratified by age (<60 vs ≥60), sex (male vs female), race (white, black or other), Charlson-Deyo Comorbidity Index (limited to 0 for this analysis), facility type (academic vs nonacademic), insurance status (none, private, Medicaid, Medicare or other), clinical T (cT) stage and stage grouping. Tumor sizes, when documented, were stratified by quartile. 

We also calculated and compared the biologically equivalent doses (BEDs) of the radiation fractionation regimens using an α/β ratio of 10 for tumor control and stratified the group between those receiving a BED of ≥ 100Gy BED and <100Gy. We then used the Kaplan-Meier method with log-rank analysis to compare the two BED groupings to see if there was an overall survival (OS) benefit to receipt of BED ≥100 Gy. 

We performed a multivariable analysis (MVA) using Cox regression to assess for other potential confounding factors impacting OS including age (<60 vs ≥60), sex (male vs female), race (white, black or other), facility type (academic vs nonacademic), insurance status (none, private, Medicaid, Medicare or other), tumor size by overall quartile, cT stage, and BED ≥100 Gy. All statistics were performed using Statistical Package for the Social Sciences (SPSS) version 24 (IBM Corp., Armonk, NY) and a p-value of 0.05 was determined to be the threshold for significance. 

## Results

After applying the total dose and dose per fraction search constraints, we identified a cohort of 462 patients for analysis. The median age was 64 (range 19-90), 75.3% of the patients were male, and 83.5% of the patients were white. Tumor sizes, when documented (n = 440) ranged from 8 to 208 mm in the largest single dimension, with interquartile ranges of 8-24, 25-35, 36-48, and 49-208 mm. Further patient characteristics are documented in Table 1. 

**Table 1:** Patient demographics

Characteristics	No. of patients (%)
Age (years)	
<60	155 (33.5)
≥60	307 (66.5)
Sex	
Male	348 (75.3)
Female	114 (24.7)
Race	
White	386 (83.5)
Black	46 (10.0)
Other/Unknown	30 (6.5)
Tumor Size, mm (n = 440)	
8-24	112 (24.2)
25-35	116 (25.1)
36-48	106 (22.9)
49-208	106 (22.9)
cT Stage (n = 462)	
1	259 (56.1)
2	122 (26.4)
3 (unspecified)	16 (3.5)
3A	19 (4.1)
3B	40 (8.7)
4	6 (1.3)
cN Stage (n = 460)	
0	449 (97.2)
X	11 (2.4)
Stage Grouping n = 462)	
I	259 (56.1)
II	122 (26.4)
III (unspecified)	16 (3.5)
IIIA	19 (4.1)
IIIB	40 (8.7)
IIIC	6 (1.3)
Facility	
Academic	363 (78.5)
Nonacademic	99 (21.4)
Insurance	
None	22 (4.8)
Private	137 (29.7)
Medicaid	47 (10.2)
Medicare	239 (51.7)
Other/Unknown	17 (3.7)

The most common SABR fractionation regimen used to treat HCC was 10Gy x five fractions (n = 117), followed by 8Gy x five fractions (n = 82), 15Gy x three fractions (n = 82) and 16Gy x three fractions (n = 40). Other common schemes utilized were 6Gy x five fractions (n = 23), 9Gy x five fractions (n =23 ), 18Gy x three fractions (n =14), 7Gy x five fractions (n = 12), 12Gy x five fractions (n = 22), 12Gy x four fractions (n = 12), 20Gy x three fractions (n = 11), 10Gy x four fractions (n = 7), and 11Gy x five factions (n = 8). No other regimen was used to treat more than two patients in total. Overall, five fraction regimens were used 63.0% of the time (n = 291), three fraction regimens were used 32.3% of the time (n = 149) and four fraction regimens were used only 4.8% of the time (n = 22). See Table 2 for further detail on fractionation regimens for each of the 462 patients. 

**Table 2:** Fractionation regimens

Dose per fraction (cGy)	No. of patients receiving three doses	No. of patients receiving four doses	No. of patients receiving five doses	Total # Pts
600	0	0	23	23
650	0	0	1	1
700	0	0	12	12
750	0	0	2	2
800	0	0	82	82
850	0	0	1	1
900	0	0	23	23
1000	0	7	117	124
1100	0	0	8	8
1150	0	1	0	1
1200	0	12	22	34
1250	0	2	0	2
1500	82	0	0	82
1600	40	0	0	40
1650	1	0	0	1
1750	1	0	0	1
1800	14	0	0	14
2000	11	0	0	11
	149	22	291	462

**Figure 1:**
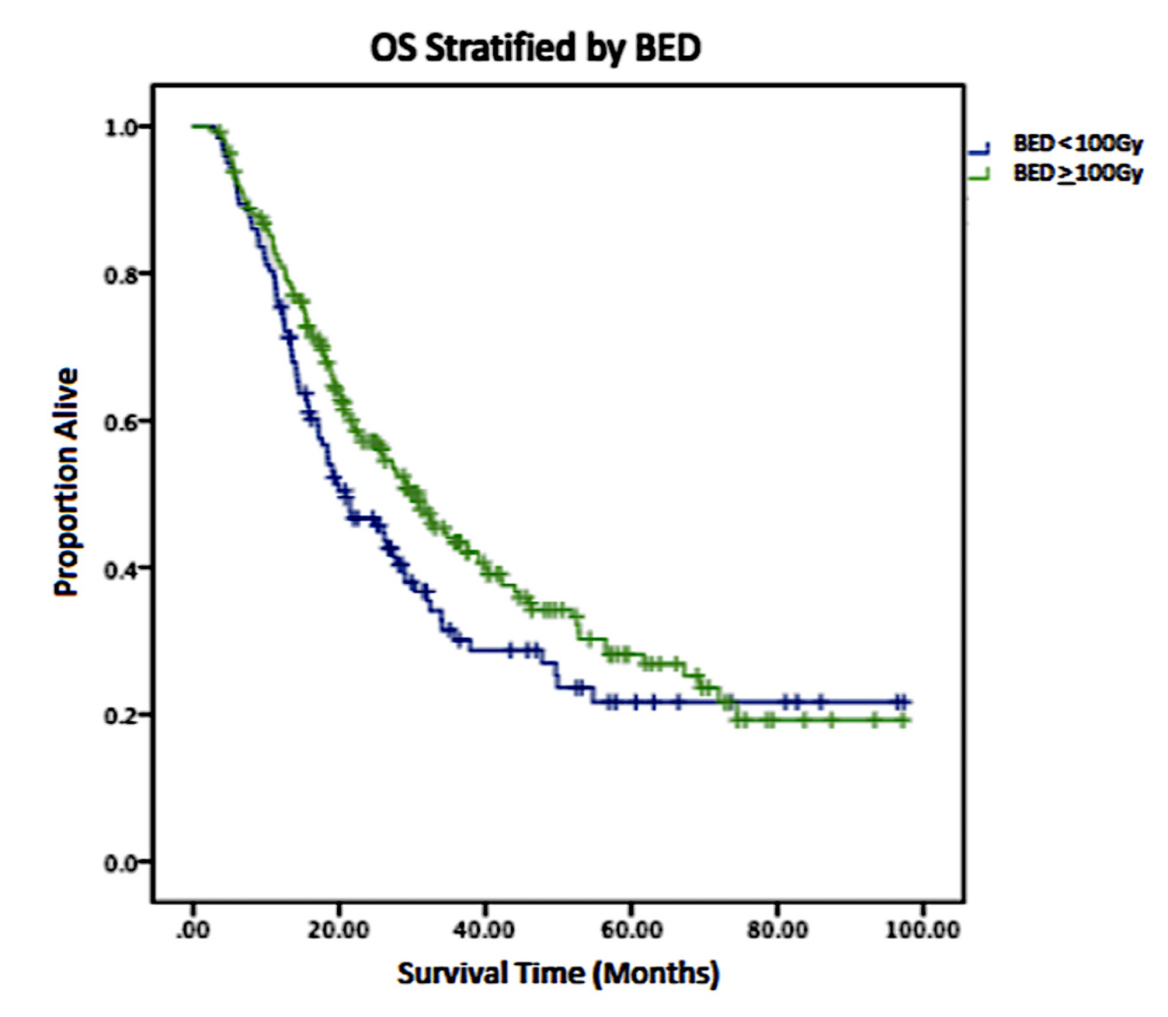
Kaplan-Meier comparison of overall survival for patients receiving BED < 100Gy compared with BED ≥ 100Gy (p = 0.062) BED, biologically equivalent dose; OS, overall survival

Three hundred and ten patients (67.1%) were treated to a total BED of at least 100Gy in three to five fraction regimens, while the remaining 152 patients (32.9%) had treatments with BED <100Gy, all in four to five fraction regimens. Details of treatment distribution and BED values are shown in Table 3 and Kaplan-Meier curve showing OS comparing BED ≥ 100 vs BED <100Gy is shown in Figure 1 (p = 0.062). Median OS was estimated to be 20.8 months with BED < 100Gy (95% CI 14.55-27.11) and 30.8 months (95% CI 25.25-32.08) with BED ≥ 100Gy. two-year and four-year OS were 46.7% and 27.0% with BED < 100Gy vs 57.1%, and 34.1% with BED ≥ 100Gy. 

**Table 3:** Details of fractionation regimens

Regimen	Number of patients	BED range (Gy, α/β = 10)
6-7.5Gy x 5	38	48-65.6
8-9.5Gy x 5	106	72-92.6
10-12Gy x 5	147	100-132
10-11.5Gy x 4	8	80-98.9
12-13Gy x 4	14	105.6-112.5
15-17.5Gy x 3	124	112.5-144.4
18-20Gy x 3	25	151.2-180
Total BED <100	152	48-98.9
Total BED ≥100	310	100-180

On MVA, BED ≥ 100Gy was not significantly associated with improved OS, with a hazard ratio (HR) of 0.85 (95% CI 0.64-1.14, p =0.28). The factors significantly associated with worse survival were tumor size in the largest quartile (HR 2.20, 95% CI 1.44-3.35, p < 0.0001 compared to the 1st), cT3a disease (HR 2.47, 95% CI 1.47-4.16, p = 0.001 compared to T1). Private insurance was associated with improved survival compared with no insurance (HR 0.49, 95% CI 0.26-0.90, p =0.02). Details of MVA are listed in Table 4. 

**Table 4:** Multivariable analysis for overall survival BED, biologically equivalent dose

Characteristic	Hazard ratio (95% CI, p value)
BED	
<100	1
≥100	0.86 (0.64-1.14, 0.29)
Age	
<60	1
≥60	1.25 (0.87-1.80, 0.23)
Sex	
Male	1
Female	0.83 (0.60-1.16, 0.28)
Race	
White	1
Black	0.81 (0.49-1.33, 0.40)
Other/Unknown	0.68 (0.36-1.27, 0.22)
Tumor Size (mm)	
8-24	1
25-35	1.434 (0.95-2.18, 0.08)
36-48	1.35 (0.89-2.04, 0.15)
49-208	2.12 (1.39-3.23, <0.0001)
cT Stage	
1	1
2	1.34 (0.98-1.84, 0.07)
3	1.28 (0.71-2.31, 0.41)
4	2.47 (1.47-4.16, 0.001)
Facility	
Academic	1
Nonacademic	1.14 (0.81-1.59, 0.46)
Insurance	
None	1
Private	0.50 (0.27-0.91, 0.03)
Medicaid	0.65 (0.32-1.29, 0.21)
Medicare	0.58 (0.31-1.05, 0.07)
Other/Unknown	0.60 (0.25-1.48, 0.27)

## Discussion

The preferred treatment of choice for patients with HCC is surgical resection or transplant. However, a significant percentage of patients are not suitable surgical candidates. These patients may be offered targeted therapy, selective yttrium-90 internal radiation, TACE and/or regional tumor ablation such as radiofrequency or microablation [9]. Historically, the use of external beam radiation was limited by collateral damage to normal liver parenchyma. In recent years, the development of partial organ irradiation using 3D conformal radiation therapy led to increased interest in the use of EBRT in inoperable HCC cases [10]. More recently, SABR has led to further increased interest and application of EBRT due to its high target accuracy and steep dose gradient, which in turn allows for improvement in local control without increased risk of toxicity [11,12].

Interest in SABR increased as studies proving its safety and efficacy began to be published. In the past decade, numerous retrospective studies have demonstrated great outcomes with SABR, including a systematic review and meta-analysis of SABR for HCC [13-15]. In the study, Qi et al. not only reported a local control rate of 87% at the longest duration of complete follow-up and three-year OS of 58% with only a 4.9% rate of grade > 3 toxicities following SABR [15]. Though there has not been a large-scale prospective, randomized trial for the treatment of HCC with SABR, there have been several smaller, prospective studies confirming its safety and efficacy in a variety of clinical settings [6,7,16-20]. Andolino et al. reported 90% two-year LC and 67% two-year OS in 60 patients with liver-confined HCC that underwent SABR [17]. Kang et al. studied the use of SABR for local salvage following incomplete TACE in 50 patients as part of the phase II trial and reported a 94.6% two-year LC and 68.7% two-year OS [19]. And, Huang et al. studied SABR specifically in recurrent HCC, finding a two-year in-field failure-free rate of 75.1% and OS of 64% [6]. It should be noted that these studies utilized a variety of doses and fractionations with no clear optimal fractionation scheme. 

To date, there has not been a study that showed clear benefit with a particular dose and fractionation [5-7,17,19-22]. For example, Andolino et al. evaluated 60 patients that received anywhere from 24Gy to 48Gy in three to five fractions, Wahl et al. evaluated 63 patients that received anywhere from 27Gy to 60Gy in three to five fractions, and Buckstein et al at Mt. Sinai delivered 24-50Gy in three to five fractions [17,21,22]. Jang et al., recognizing the diversity in SABR regimen for HCC, took a slightly different approach and evaluated only those patients that received three fractions but categorized them based on the total dose of >54Gy, 45-54Gy, and <45Gy [20]. Similarly, Kang et al. treated 56 lesions in 47 patients with 42-60Gy in three fractions [19]. In our study, we analyzed a total of 462 patients treated with SABR in three to five fractions for HCC between the years 2004 and 2014. Per our findings, the most common fractionation regimen is 50Gy delivered in five fractions with other common fractionations being 40Gy in five fractions and 45-48Gy in three fractions. 

In the present study, when we compared patients that received BED of < 100Gy with those receiving BED ≥100Gy, we found no significant difference in OS (p = 0.062). Median OS was estimated to be 20.8 months with BED < 100Gy (95% CI 14.55-27.11) and 30.8 months (95% CI 25.25-32.08) with BED ≥ 100Gy. The two-year and four-year OS were 46.7% and 27.0% with BED < 100Gy vs 57.1%, and 34.1% with BED ≥ 100Gy. In a majority of the studies, determination of the fractionation regimen and thus BED has been left to the judgement of the treating physician. Unfortunately, data and factors that the decision would be based on are not available within the NCDB database. 

A few prospective studies did have a set protocol to determine the optimal fractionation regimen. For example, Takeda et al. prescribed based on Child-Pugh class and dose to normal tissue, delivering 35Gy in five fractions to patients with either Child-Pugh B disease or Child Pugh Class A disease with >20% of normal liver receiving >20Gy and all others received 40Gy in five fractions [23]. Cardenes et al. carried out a feasibility trial, prescribing 48Gy at 16Gy/fraction to patients with Child-Pugh Class A and 40Gy at 8Gy/fraction to patients with Child-Pugh Class B [24]. Similarly, Moon et al. delivered 45Gy in three fractions to patients with Child Pugh Class A with dose de-escalation to meet predetermined dose constraints such as at least 700cGy liver <15Gy and D35% of liver <15Gy [25]. Patients with Child Pugh Class B were treated with 35Gy in five fractions with dose de-escalation to meet stricter dose constraints such as D50% of liver <15Gy. Ultimately, total dose ranged from 27.5Gy to 45Gy in three to five fractions. As these studies indicate, the ultimate dose and thus BED depend on multiple patient and tumor characteristics. As the most common of these characteristics were liver function and normal liver dose constraints, neither of which are available within the NCDB, it is difficult to conclude anything definitive regarding the dose-response relationship based on NCDB data.

Limitations of this study, in addition to that which was noted above, include those inherent to most, if not all studies, which extract data from national databases. This includes ascertainment bias and lack of data on local control, disease-free survival, cause of death, or toxicity. In regards to data on toxicity, there have been numerous previous studies that have already evaluated toxicity following SABR. Louis et al. specifically looked at patients that received 45Gy in three fractions delivered over 10-12 days and noted 8% grade 3 acute toxicity rate, no acute grade 4 toxicities, and minimal late toxicity [26]. And in another meta-analysis, toxicities > grade 3 were noted to be 4.9% [15]. Limitations specific to the disease site include lack of imaging access and lack of data on Childs Pugh score, prior treatments if any, and dose volume histograms (DVH), all of which make it difficult to evaluate why certain doses were given in various scenarios and utilization of SABR in the context of other components of an individual’s treatment that could also attribute to his or her outcomes. Thus, the reported results do not allow for any definitive conclusions but rather provide basis and direction for further studies. 

However, this study does provide a large dataset from which we can gain a frame of reference of the diverse dosing regimens and treatments offered for liver SABR. Larger, ideally prospective, studies are needed to better standardize the planned radiation dose, fractionation schedule, and whether or not there is a dose-response relationship that improves local control and/or survival.

## Conclusions

In conclusion, though the use and application of SABR for the treatment of unresectable HCC continues to expand in recent years, multiple SABR fractionation schemes are still utilized at this time. The most commonly used fractionations are 10Gy x five fractions followed by 8Gy x five fractions and 15Gy x three fractions. And per our analysis, BED of 100Gy or greater is not correlated with improved survival in this limited patient sample and with the patient exclusion criteria used in this study. Further studies are needed to best identify the optimal SABR dose and fractionation.
